# Genome Sequence of *Clostridium paraputrificum* 373-A1 Isolated in Chile from a Patient Infected with *Clostridium difficile*

**DOI:** 10.1128/genomeA.01178-16

**Published:** 2016-11-03

**Authors:** Enzo Guerrero-Araya, Angela Plaza-Garrido, Fernando Díaz-Yañez, Marjorie Pizaro-Guajardo, Sandro L. Valenzuela, Claudio Meneses, Fernando Gil, Eduardo Castro-Nallar, Daniel Paredes-Sabja

**Affiliations:** a Microbiota-Host Interactions and Clostridia Research Group, Departamento de Ciencias Biológicas, Facultad de Ciencias Biológicas, Universidad Andrés, Bello, Santiago, Chile; b Center for Bioinformatics and Integrative Biology, Facultad de Ciencias Biológicas, Universidad Andrés, Bello, Santiago, Chile; c Centro de Biotecnología Vegetal, Facultad de Ciencias Biológicas, Universidad Andrés, Bello, Santiago, Chile; d FONDAP Center for Genome Regulation, Santiago, Chile

## Abstract

*Clostridium paraputrificum* is a gut microbiota member reported in several cases of bacteremia and coinfections. So far, only one genome sequence of a *C. paraputrificum* (AGR2156) isolate is available. Here, we present the draft genome of *C. paraputrificum* strain 373-A1, isolated from stools from a patient with *C. difficile* infection.

## GENOME ANNOUNCEMENT

*Clostridium paraputrificum* is a Gram-positive, endospore-forming, strictly anaerobic, and chitinolytic bacterium (1). Few studies have addressed features of *C. paraputrificum*. Early work associated *C. paraputrificum* with an increased risk of colon cancer (2). *C. paraputrificum* has also been isolated from healthy individuals (3), as well as from cases of bacteremia (4) and patients with AIDS (5). *C. paraputrificum* contributes to colonic epithelium maturation and development (6); however, its role in infectious diseases remains unclear.

Genomic data for strain 373-A1 was generated using Illumina technology (7) on an Illumina MiSeq platform (600 incorporated cycles; 2 × 300 bp). Library preparation was carried out according to the TruSeq DNA kit, generating 3,165,742 paired-end reads and 1.47 GB of information. Next, raw data were filtered (>Q20) and merged (minimum overlapping of 20 bp) in PEAR version 0.9.8 (8). The resulting set of reads was recleaned using Pathoscope version 2.0.6 (9) in order to obtain only the reads mapping against bacterial genomes. Data were assembled using SPAdes version 3.5.0 (10) with an auto cutoff coverage. The final assembly contained 41 contigs, and an *N*_50_ equal to 259,466 bp with a mean of coverage of 472×.

The genome annotation was performed with the NCBI Prokaryotic Genome Annotation Pipeline (11) and this annotation was saved in GenBank (MAPZ01). The assembly of the draft genome sequence consists of 41 contigs amounting to 3,488,595 bp with a G+C content of 29.8%. Of the 3,351 predicted genes, 3,220 were protein-coding genes, and 106 RNAs and 24 rRNA operons were identified. The majority of the protein-coding genes (60.19%) were assigned a putative function, while the remaining ones were annotated as hypothetical proteins.

For a comparison, the assembly report ASM42402v1 (*C. paraputrificum* AGR2156) was used. The genome of *C. paraputrificum* 373-A1 is smaller than *C. paraputrificum* AGR2156 (i.e., 3,488,595 bp versus 3,561,289 bp, respectively). *C. paraputrificum* 373-A1 had fewer predicted genes and genes encoding putative proteins (i.e., 3,351 genes and 3,220 proteins) than *C. paraputrificum* AGR2156 (i.e., 3,457 genes and 3,345 proteins). On the other hand, the G+C content was similar in both strains, with 29.8% and 29.6% for *C. paraputrificum* 373-A1 and *C. paraputrificum* AGR2156, respectively. When we focus on the number of contigs and *N*_50_ values, *C. paraputrificum* 373-A1 has 41 contigs with an *N*_50_ value of 259,466, in contrast with *C. paraputrificum* AGR2156, which has 32 contigs with an *N*_50_ value of 320,228, which indicates the high quality of our genome sequence.

This draft sequence provides a new repertoire of genes and genome information for this strain of the opportunistic *C. paraputrificum*.

### Accession number(s).

The *C. paraputrificum* 373-A1 genome has been deposited in GenBank under the accession number MAPZ00000000.
